# Fulminant Hepatitis Revealing Carob Intoxication: A Case Report

**DOI:** 10.7759/cureus.83882

**Published:** 2025-05-11

**Authors:** Mohammed Amine Oulalite, Hamza Mimouni, Meriem Jartit, Housam Bkiyar, Brahim Housni

**Affiliations:** 1 Department of Anesthesia and Reanimation, Faculty of Medicine and Pharmacy, Mohammed VI University Hospital, Mohammed First University, Oujda, MAR; 2 Department of Neurology, Regional Hospital Center Al Farabi, Oujda, MAR

**Keywords:** ceratonia siliqua, fulminant hepatitis, intoxication, liver failure, toxic hepatitis

## Abstract

A 34-year-old man with a history of obsessive-compulsive disorder was admitted with febrile altered consciousness and generalized mucocutaneous jaundice. His condition was attributed to excessive consumption of Ceratonia siliqua (also known as carob), which he believed had digestive benefits. On examination, he had an altered consciousness with a Glasgow Coma Scale evaluation at 8/15, was hemodynamically stable, and exhibited adequate respiratory function. Laboratory findings revealed severe hypoglycemia, hepatocellular failure with marked cytolysis, and a critically low prothrombin level, while viral serologies were negative and renal function remained normal. A cranial CT scan showed no abnormalities. In the absence of an identifiable cause for fulminant hepatitis, carob intoxication was suspected. The patient received mechanical ventilation, supportive care with 10% glucose infusion, vitamin K therapy, prophylactic ceftriaxone, laxatives, and plasmapheresis for five days. Despite intensive management, his condition deteriorated, and he succumbed to acute liver failure.

## Introduction

The carob tree has been valued for thousands of years by the inhabitants of Mediterranean countries for its sweet, floury pulp. Believed to have originated in Syria, it was cultivated by the Egyptians, who used carob flour to stiffen the wrappings of their mummies. In Morocco, Berbers traditionally utilized carob for its medicinal properties, particularly its high fiber content; its fruit was dissolved in a hot liquid to treat diarrhea [1]. In traditional medicine, carob is known for its anticholesterolemic, antidiarrheal, and gastroprotective properties. However, excessive consumption can lead to toxicity [2,3]. We report a case of intoxication in a young man admitted to the intensive care unit (ICU) with multi-organ failure following the ingestion of a large quantity of carob powder dissolved in water. He had consumed it for therapeutic purposes to alleviate functional colopathy. This case highlights a rare instance of carob intoxication, warranting further discussion on its potential risks.

## Case presentation

A 34-year-old man with a history of obsessive-compulsive disorder and functional colopathy, managed with natural and plant-based remedies, was admitted to the ICU for febrile encephalopathy, accompanied by generalized cutaneous and mucosal jaundice. According to his family, he had significantly increased his intake of carob powder, consuming approximately five to six heaping tablespoons daily (estimated 100-120 g) starting six days prior to admission, based on a personal belief in its digestive benefits.

On examination, he was unconscious with a Glasgow Coma Scale (GCS) score of 8/15, exhibited miosis, and was hemodynamically stable, with a blood pressure of 120/75 mmHg, a heart rate of 78 beats per minute, a respiratory rate of 33 breaths per minute, and an SpO₂ of 90%. He was febrile at 38.5°C (101.3°F) with generalized jaundice, while abdominal and cardiopulmonary examinations were unremarkable.

Laboratory tests revealed normal blood counts and renal function but showed severe liver dysfunction with significant cytolysis: aspartate aminotransferase (AST) of 250 IU/L (normal: 10-40 IU/L), alanine transaminase (ALT) of 290 IU/L (normal: 7-45 IU/L), alkaline phosphatase (ALP) of 534 IU/L (normal: 35-130 IU/L), and a prothrombin time of 25% (normal: 70-100%), indicating coagulopathy and cholestasis. Extensive serological testing for hepatitis A, B, and C, cytomegalovirus (CMV), Epstein-Barr virus (EBV), and human immunodeficiency virus (HIV) was negative, as were autoimmune markers, including anti-smooth muscle antibody, anti-actin antibody, anti-nuclear antibody (ANA), and anti-soluble liver antigen (SLA) antibody (Table 1).

**Table 1:** Table showing laboratory investigations CMV: cytomegalovirus, EBV: Epstein-Barr virus, HIV: human immunodeficiency virus

Test	Result	Normal Range
Aspartate Aminotransferase (AST)	250 IU/L	10-40 IU/L
Alanine Aminotransferase (ALT)	290 IU/L	7-45 IU/L
Alkaline Phosphatase (ALP)	534 IU/L	35-130 IU/L
Prothrombin Rate	25%	70-100%
White Blood Cell (WBC)	6,500/µL	4,000-11,000/µL
Red Blood Cell (RBC)	5.0 million/µL	4.5-5.9 million/µL
Platelets	250,000/µL	150,000-450,000/µL
Creatinine	1.0 mg/dL	0.6-1.2 mg/dL
Urea	18 mg/dL	7-20 mg/dL
Hepatitis A	Negative	Negative
Hepatitis B	Negative	Negative
Hepatitis C	Negative	Negative
CMV	Negative	Negative
EBV	Negative	Negative
HIV	Negative	Negative
Autoimmune Markers	Negative	Negative

A brain computed tomography scan was performed on admission and revealed no abnormalities. The cerebral parenchyma was of normal density, with preserved grey-white matter differentiation, no evidence of cerebral edema, mass effect, hemorrhage, or ischemic lesions. Ventricular size and configuration were normal, and no abnormal enhancement was noted post-contrast administration.

Based on clinical and laboratory findings, a diagnosis of acute fulminant hepatitis was made. The patient was promptly intubated and placed on mechanical ventilation due to declining consciousness and respiratory compromise. Supportive management included intravenous dextrose 10% infusion to maintain normoglycemia and prevent hypoglycaemia secondary to hepatic dysfunction, along with parenteral vitamin K (10 mg daily) to correct coagulopathy. Empirical broad-spectrum antibiotic therapy (ceftriaxone and metronidazole) was initiated to prevent potential infections. Lactulose was administered via nasogastric tube to reduce ammonia levels and reduce hepatic encephalopathy. Additionally, therapeutic plasmapheresis was performed daily for five consecutive days to manage hepatic failure and support detoxification. Despite aggressive supportive care, his condition progressively worsened, and he died from complications of hepatic failure seven days after ICU admission.

## Discussion

The carob tree (Ceratonia siliqua L.) is one of the most common medicinal plants in the Mediterranean region, belonging to the Leguminosae (syn. Fabaceae) subfamily of the Caesalpinaceae genus. Traditionally, it has been cultivated for its ethnopharmacological properties. The plant contains various phytoconstituents, including phenolic acids, flavonoids, tannins, and alkaloids. Recent studies have demonstrated that extracts from this plant exhibit anti-inflammatory, antibacterial, antifungal, anti-diarrheal, antioxidant, and antiproliferative activities [4-9]. The high concentration of phenolic compounds in C. siliqua is directly related to its antioxidant activity, as reported by El-Hajaji et al. [10]. Additionally, studies have shown that the leaves of C. siliqua inhibit the enzymes glucosidase and amylase, suggesting potential anti-diabetic effects [11].

Gulay et al. [12] investigated the toxicological effects of C. siliqua on male New Zealand White rabbits. In their study, rabbits in the treatment group were administered 10 cc of carob remedy made by boiling the carob fruit. Over the course of a seven-week investigation, no toxicological symptoms or fatalities related to the carob extract were observed. However, no extensive toxicological studies have been conducted to evaluate the effects of this plant at excessive doses.

Our clinical case represents the first documented instance of C. siliqua intoxication in the literature. The patient, suffering from obsessive-compulsive disorder, consumed large quantities of the plant, believing it to have digestive benefits. An extensive etiological investigation of the fulminant hepatitis the patient presented with revealed no obvious causes, including viral, medication-related, toxic (other than the consumption of carob), or autoimmune origins. Thus, intoxication with C. siliqua was considered the likely etiology.

The patient received symptomatic treatment, which included prevention of hypoglycemia, the use of laxatives, vitamin K therapy to prevent coagulation disorders, antibiotic therapy, and plasmapheresis. Unfortunately, as liver transplantation was not available at our center, the patient's condition worsened, and he eventually passed away.

## Conclusions

This case highlights the possible risks associated with the excessive consumption of C. siliqua, a medicinal plant commonly used in the Mediterranean region. Although generally considered safe and traditionally valued for its therapeutic properties, including antidiarrheal and gastroprotective effects, our report suggests a possible adverse effect resulting from overconsumption. Despite the plant’s well-documented benefits, this case underscores the importance of caution when using carob for medicinal purposes at high doses. The lack of extensive toxicological studies on its effects at excessive intake further emphasizes the need for additional research to better define its safety profile. Clinicians should remain alert to the possibility of C. siliqua intoxication and consider it in the differential diagnosis of unexplained acute liver failure.
